# Development of a novel equine influenza virus live-attenuated vaccine

**DOI:** 10.1016/j.virol.2018.01.005

**Published:** 2018-03

**Authors:** Laura Rodriguez, Stephanie Reedy, Aitor Nogales, Pablo R. Murcia, Thomas M. Chambers, Luis Martinez-Sobrido

**Affiliations:** a Department of Microbiology and Immunology, University of Rochester, Rochester, NY, United States; b Department of Veterinary Science, Gluck Equine Research Center, University of Kentucky, Lexington, KY, United States; c MRC-University of Glasgow Centre for Virus Research, Glasgow, United Kingdom

**Keywords:** Influenza A virus (IAV), Equine influenza virus (EIV), Live-attenuated influenza vaccine (LAIV), Master donor virus (MDV), Reverse genetics techniques, Temperature sensitive (ts), Attenuated (att)

## Abstract

H3N8 equine influenza virus (EIV) is an important and significant respiratory pathogen of horses. EIV is enzootic in Europe and North America, mainly due to the suboptimal efficacy of current vaccines. We describe, for the first time, the generation of a temperature sensitive (ts) H3N8 EIV live-attenuated influenza vaccine (LAIV) using reverse-genetics approaches. Our EIV LAIV was attenuated (att) *in vivo* and able to induce, upon a single intranasal administration, protection against H3N8 EIV wild-type (WT) challenge in both a mouse model and the natural host, the horse. Notably, since our EIV LAIV was generated using reverse genetics, the vaccine can be easily updated against drifting or emerging strains of EIV using the safety backbone of our EIV LAIV as master donor virus (MDV). These results demonstrate the feasibility of implementing a novel EIV LAIV approach for the prevention and control of currently circulating H3N8 EIVs in horse populations.

## Highlights

• We have generated an equine influenza virus (EIV) live-attenuated influenza vaccine (LAIV) using reverse genetics approaches.
• Our EIV LAIV replicates efficiently at permissive (33°C) temperatures, important for vaccine production.
• The EIV LAIV is attenuated in vivo, important for its implementation as a LAIV.
• Our EIV LAIV is able to induce protection against EIV wild-type challenge in both mice and horses.
• This is the first description of an EIV LAIV based on reverse genetics approaches.

## 1 Introduction

Influenza A viruses (IAVs) are enveloped viruses in the *Orthomyxoviridae* family that contain a segmented genome made of eight single-stranded RNA molecules of negative polarity (Palese and Shaw, 2007). IAVs are classified by subtypes based on the antigenicity of the two major membrane glycoproteins: hemagglutinin (HA) and neuraminidase (NA) (Palese and Shaw, 2007). Equine influenza, caused by equine influenza virus (EIV), is the most common and important respiratory infectious disease of horses. The H3N8 subtype of EIV was first reported from infected horses in Florida in 1963 (Waddell et al., 1963). At the end of the 1980s, H3N8 EIV diverged phylogenetically and antigenically into the American and Eurasian lineages (Daly et al., 1996). The American lineage evolved into Florida, Kentucky and South American sublineages (Lai et al., 2001), and the Florida sublineage has further diverged into the clades 1 and 2 that continue circulating nowadays (Bryant et al., 2009, Murcia et al., 2011). Currently, viruses from the sublineage Florida clade 1 are considered enzootic in the United States (US) but have also produced outbreaks in other parts of the world (Alves Beuttemmuller et al., 2016, Cowled et al., 2009, Woodward et al., 2014; Yamanaka et al., 2008), while the clade 2 viruses of the Florida sublineage are predominant in Europe and Asia (Fougerolle et al., 2017, Qi et al., 2010, Rash et al., 2017, Virmani et al., 2010, Yondon et al., 2013). Based on international surveillance studies, the World Organization for Animal Health (OIE, Office International des Epizooties) recommends including representative viruses from both the sublineage Florida clades 1 and 2 in the composition of H3N8 EIV vaccines (OIE, 2017).

Vaccination is the most effective strategy, alongside isolation, movement restriction and basic biosecurity measures, to prevent H3N8 EIV infections or to limit their consequences (Pica and Palese, 2013, Wong and Webby, 2013). Despite the development and commercialization of vaccines for almost five decades, H3N8 EIV is still circulating and considered endemic in numerous countries around the World, including the US (Cullinane et al., 2010, Paillot, 2014, Paillot et al., 2016). Different vaccine strategies have been available for decades for the control of EIV in horses. These include, mainly, influenza inactivated (IIV) and live-attenuated (LAIV) vaccines. Several vaccination studies have showed that adjuvanted IIVs administered intramuscularly induce humoral immunity, mostly by inducing neutralizing antibodies against the viral HA protein, but are comparatively poor inducers of cellular immunity (Belongia et al., 2009, Osterholm et al., 2012, Paillot, 2014). There is a wide panel of EIV IIVs, but the large majority of them do not contain representative strains of both clades 1 and 2 of the Florida sublineage of H3N8 EIV as recently recommended by the OIE. LAIVs are administered intranasally, mimicking the natural route of viral infection, and are able to induce both cellular and humoral immune responses, providing better immunogenicity and protection than IIVs (Belshe et al., 2007, Gorse et al., 1991, Hoft et al., 2011, Paillot, 2014). The only currently available H3N8 EIV LAIV, Flu Avert I.N. (Merck), was developed by passaging the A/equine/Kentucky/1/1991 H3N8 (Kentucky lineage) in embryonated chicken eggs at gradually reduced temperatures to generate a temperature sensitive (ts) variant that replicates efficiently at low temperatures (cold-adapted, ca) (Wilson and Robinson, 2000, Youngner et al., 2001). It has been shown that Flu Avert I.N. induced homologous (Lunn et al., 2001) and heterologous (Chambers et al., 2001) protection against H3N8 EIVs circulating in the 1990s. Thus, if the circulating EIV matches the virus in the vaccine (A/equine/Kentucky/1/1991), Flu Avert I.N. can confer better protection against disease caused by EIVs than the IIV counterparts, inducing faster production of antibodies and broader immune responses (Cullinane et al., 2010, Paillot, 2014, Paillot et al., 2016). For this reason, LAIVs are ideal for their use to prevent and control EIV infections (Cullinane et al., 2010, Paillot, 2014, Paillot et al., 2016). However, although other LAIVs are updated yearly, Flu Avert I.N. has not been updated or modified to match currently circulating EIV strains. Therefore, and because of the ability of influenza virus to undergo antigenic drift, there is not a good match of surface antigens between contemporary EIV strains and the virus present in Flu Avert I.N. The antigenic disparity between the virus in Flu Avert I.N. and currently circulating EIV strains likely results in a significantly lower vaccination impact (Cullinane et al., 2010, Paillot, 2014) and partial protection (Park et al., 2009, Yates and Mumford, 2000). Moreover, Flu Avert I.N. does not contain any representative Florida sublineage clade 1 and 2 H3N8 EIV strains, which is recommended by the OIE to be included in H3N8 EIV vaccines (Paillot et al., 2016).

In order to develop an updated and more effective LAIV for the treatment of currently circulating EIV strains, we used the same strategy that we have recently implemented for the development of LAIVs against H3N8 and H3N2 canine influenza viruses (CIVs) (Nogales et al., 2016b, Rodriguez et al., 2017c). We introduced in the polymerase basic 2 (PB2) and polymerase basic 1 (PB1) viral proteins of A/equine/Ohio/1/2003 H3N8 (Florida sublineage clade 1) the mutations responsible for the ts, ca and att phenotype of A/Ann Arbor/6/60 H2N2 LAIV (Cox et al., 1988, Snyder et al., 1988), the master donor virus (MDV) of the human LAIV (FluMist, MedImmune) and assessed its safety and efficacy in both mice and horses. This is the first description of a ts and att H3N8 EIV LAIV obtained by reverse-genetics technology.

## 2 Materials and methods

### 2.1 Cells and viruses

Human embryonic kidney 293 T cells (293 T; ATCC CRL-11268), Madin-Darby canine kidney cells (MDCK; ATCC CCL-34) and equine dermal cells (E. Derm NBL-6; ATCC CCL-57) were grown in Dulbecco's modified Eagle's medium (DMEM; Mediatech, Inc.) supplemented with 10% fetal bovine serum (FBS), and 1% PSG (penicillin, 100 units/ml; streptomycin 100 µg/ml; L-glutamine, 2 mM) at 37°C with 5% CO_2_ (Nogales et al., 2014b).

Recombinant wild-type (WT) and live attenuated (LAIV) H3N8 EIVs were generated using A/equine/Ohio/1/2003 plasmid-based reverse techniques (Martinez-Sobrido and Garcia-Sastre, 2010) and grown in MDCK cells at 33°C. The commercially available A/equine/Kentucky/1/1991 H3N8 LAIV (Flu Avert I.N., Merck) was also grown in MDCK cells at 33°C. The A/equine/Kentucky/2014 H3N8, used in horse challenge experiments, was grown in embryonated hen eggs. For infections, virus preparations were diluted in phosphate buffered saline (PBS) containing 0.3% bovine albumin (BA) and 1% penicillin and streptomycin (PS) (PBS/BA/PS). After 1 h viral adsorption at room temperature (RT), MDCK cells were maintained with post-infection (p.i.) DMEM media supplemented with 0.3% BA, 1% PSG, and 1 μg/ml of N-tosyl-L-phenylalanine chloromethyl ketone (TPCK)-treated trypsin (Sigma). Viral titers were determined by immunofocus assay (fluorescent forming units, FFU/ml) in MDCK cells at 33°C as previously described (Nogales et al., 2014b) using the anti-NP monoclonal antibody (mAb) HB-65 (ATCC HB-65, HL16-L10-4R5).

### 2.2 Plasmids

For the generation of H3N8 EIV LAIV, the PB2 and PB1 genes of A/equine/Ohio/1/2003 H3N8 were subcloned in a pUC19 plasmid (New England BioLabs) to introduce the ts mutations PB2 N265S and PB1 K391E, E581G, and A661T by site-directed mutagenesis. The presence of the introduced mutations was confirmed by sequencing. PB2- and PB1-LAIV viral segments were subcloned from pUC19 into the ambisense pDZ plasmid like the other A/equine/Ohio/1/2003 H3N8 viral genes (PB2- and PB1-WT, PA, HA, NP, NA, M and NS) for virus rescue. pDZ is an ambisense vector that contains a human RNA polymerase I promoter and a mouse terminator sequence that encodes the negative sense genomic RNA and, in opposite orientation to the polymerase I unit, contains a polymerase II transcription cassette (chicken β-actin promoter and polyA) that encode the viral proteins from the same viral gene (Chambers et al., 2009).

### 2.3 Minigenome assay

To analyze the ability of A/equine/Ohio/1/2003 H3N8 WT and LAIV polymerases to replicate and transcribe at different temperatures (33°C, 37°C, and 39°C) E. Derm cells (12-well plate format, 5 × 10^5^ cells/well, triplicates) were co-transfected in suspension, using Lipofectamine 2000 (Invitrogen), with 0.25 μg of each of the A/equine/Ohio/1/2003 H3N8 WT or LAIV ambisense pDZ-PB2 or PB2-LAIV, pDZ-PB1 or PB1-LAIV, pDZ-PA and pDZ-NP plasmids, together with 0.5 μg of a reporter minigenome (MG) viral (v)RNA-like expression plasmid encoding Gaussia luciferase (Gluc) driven by a murine RNA polymerase I promoter (mpPol-I Gluc), and 0.1 μg of a mammalian expression pCAGGS plasmid encoding Cypridina luciferase (Cluc) to normalize transfection efficiencies (Cheng et al., 2015, Nogales et al., 2016b). Cells transfected in the absence of the pDZ-NP plasmid were included as negative control and empty pDZ plasmid was used to keep the amount of transfected plasmid DNA constant in the negative control. At 48 h post-transfection, Gluc and Cluc expression levels were determined using the Biolux Gaussia and Cypridina Luciferase Assay kits (New England BioLabs) and quantified with a Lumicount luminometer (Packard). Reporter gene activation (Gluc) was normalized to that of Cluc and is reported as fold induction over the level of induction for the negative control (absence of NP). The mean values and standard deviations (SDs) were calculated and statistical analysis was performed using a two-tailed Student *t*-test with Microsoft Excel software. Data are represented as relative activity considering A/equine/Ohio/1/2003 H3N8 WT polymerase activity at each temperature as 100%.

### 2.4 Virus rescue

Viral rescue of A/equine/Ohio/1/2003 H3N8 WT and LAIV was performed as previously described (Nogales et al., 2014b). Briefly, co-cultures (1:1) of 293 T and MDCK cells (6-well plate format, 1 × 10^6^ cells/well, triplicates) were co-transfected in suspension, using Lipofectamine 2000, with 1 μg of the eight-ambisense A/equine/Ohio/1/2003 H3N8 pDZ-PB2 or PB2-LAIV, -PB1 or PB1-LAIV, -PA, -HA, -NP, -NA, -M, and -NS plasmids. At 12 h post-transfection, the medium was replaced with p.i. DMEM medium supplemented with 0.5 μg/ml TPCK-treated trypsin. Tissue culture supernatants (TCS) were collected at three days post-transfection, clarified, and used to infect fresh monolayers of MDCK cells. Then, at three days p.i., recombinant viruses were plaque purified and scaled up using MDCK cells at 33°C (Martinez-Sobrido and Garcia-Sastre, 2010).

### 2.5 Virus growth kinetics

Multicycle viral growth kinetics was assessed by infecting MDCK cells (12-well plate format, 5 × 10^5^ cells/well, triplicates) with A/equine/Ohio/1/2003 H3N8 WT and LAIV at a multiplicity of infection (MOI) of 0.001. MDCK cells were also infected with Flu Avert I.N. using an MOI of 0.001 as a control because it constitutes a ts H3N8 EIV. After 1 h viral adsorption at RT, infection medium was replaced by p.i. DMEM medium supplemented with 0.5 μg/ml TPCK-treated trypsin and plates were incubated at different temperatures (33°C, 37°C and 39°C). TCS were collected at the indicated times p.i. and viral titers in TCS were determined by immunofocus assay (FFU/ml) in MDCK cells as indicated before (Nogales et al., 2014b). The mean values and SDs were calculated using Microsoft Excel software.

### 2.6 Plaque assay

Confluent monolayers of MDCK cells (6-well plate format, 1 × 10^6^ cells/well), were infected with the indicated viruses for 1 h at RT, overlaid with agar, and incubated at 33°C, 37°C, or 39°C. At three days p.i., the cells were fixed for 1 h at RT with 4% paraformaldehyde (PFA) and the overlays were removed. Cells were then permeabilized (0.5% Triton X-100 in PBS) for 15 min at RT and prepared for immunostaining using the anti-NP mAb HB-65 and vector kits (Vectastain ABC vector kits and DAB HRP substrate kit; Vector) according to the manufacturer's specifications (Nogales et al., 2014a, Nogales et al., 2014b, Nogales et al., 2016a).

### 2.7 Mouse experiments

Six-to-eight-week-old female C57BL/6 mice were purchased from the National Cancer Institute (NCI) and maintained in the animal care facility at the University of Rochester under specific pathogen-free conditions. All mouse protocols were approved by the University Committee of Animal Resources and complied with the recommendations in the Guide for the Care and Use of Laboratory Animals of the National Research Council (National Research Council (U.S.) et al., 2011). To evaluate the *in vivo* attenuation of EIV LAIV, six mice were anesthetized intraperitoneally (i.p.) with 2,2,2-tribromoethanol (Avertin; 240 mg/kg of body weight) and then inoculated intranasally (i.n.) with 30 μl of a virus preparation containing 10^5^ FFU of EIV WT or LAIV diluted in PBS (Rodriguez et al., 2017a). As a control, a group of mice (N = 6) was also inoculated i.n. with 10^5^ FFU of Flu Avert I.N. Virus replication was determined by measuring viral titers in the lungs and nasal mucosa of infected mice at days 2 (N = 3) and day 4 (N = 3) p.i. To that end, mice from each group were euthanized by administration of a lethal dose of Avertin and exsanguination, and the lungs and nasal mucosa were recovered and homogenized (Rodriguez et al., 2017a). Virus titers in both tissues were determined by immunofocus assay (FFU/ml) as indicated before (Nogales et al., 2014b, Rodriguez et al., 2017a).

For the vaccination and challenge experiments, 6–8-week-old female C57BL/6 mice (N = 6) were anesthetized and vaccinated i.n. with PBS or 10^3^ FFU of EIV WT, LAIV or Flu Avert I.N. (A/equine/Kentucky/1/1991 H3N8 LAIV). At fourteen days post-vaccination, mouse sera were collected by submandibular bleeding to evaluate the presence of total antibodies by enzyme-linked immunosorbent assay (ELISA) and neutralizing antibodies by hemagglutination inhibition (HAI) assay. Twenty-four hours after mice bleeding, mice were challenged i.n. with 10^5^ FFU of A/equine/Ohio/1/2003 H3N8 WT. After challenge, viral replication in mouse lungs was evaluated at days 2 (N = 3) and 4 (N = 3) p.i. as described above (Rodriguez et al., 2017a).

### 2.8 Horse experiments

Male and female one-to-two-year-old horses of mixed breed (mainly Standardbred-quarter horse crosses) were used. Horses were raised at the University of Kentucky's Maine Chance Farm as part of a closed herd, and had not been previously vaccinated for EIV. All horses were seronegative for EIV H3N8, as measured by hemagglutination inhibition assay (HAI) prior to the start of the study (data not shown). Horse experiments were approved by the University of Kentucky's Institutional Animal Care and Use Committee (Protocol no. 2007-0153). To evaluate the *in vivo* attenuation of A/equine/Ohio/1/2003 H3N8 LAIV, horses (N = 4) were inoculated by i.n. intubation with 2 ml of a virus preparation containing 4 × 10^8^ FFU of A/equine/Ohio/1/2003 H3N8 LAIV diluted in PBS. This dose, the maximum available and similar to that used in the pilot studies of the Flu Avert I.N. LAIV by Heska Corp. (Wilson and Robinson, 2000), was chosen so as to provide the greatest likelihood of revealing any clinical signs induced by the LAIV. Viral attenuation was tested daily by the observation of clinical signs, measurement of rectal temperatures and by determining the presence of virus in nasopharyngeal swabs that were taken prior to vaccination (day 0) and daily for three days thereafter. The presence of virus in nasal swabs was determined by quantitative (q)RT-PCR as described before (Lu et al., 2009).

For the vaccination and challenge experiments, one-to-two years-old horses (N = 4) were vaccinated by i.n. inoculation with 2 ml of a virus preparation containing 4 × 10^8^ FFU of A/equine/Ohio/1/2003 H3N8 LAIV. Another group of horses (N = 2) were used as a control (unvaccinated). To avoid exposure of control horses to shed EIV LAIV, the latter were pastured separately. At 27 days post-vaccination, all horses (N = 6) were brought into a BSL-2 isolation barn. The challenge virus, a heterologous Florida clade 1 EIV strain, A/equine/Kentucky/2014 H3N8, was aerosolized using a DeVillbis Ultra-Neb 99 nebulizer, and pumped into a tented stall (37.5 m^3^) to a density of 1 × 10^7^ 50% egg infectious dose (EID_50_) units per m^3^, where it was inhaled by the horses for 45 min (Mumford et al., 1990, Townsend et al., 2001). The challenge dose of virus was similar to that we have used in previous experimental infection of horses (Lunn et al., 2001). Horses were observed daily thereafter and rectal temperatures, clinical signs, and nasopharyngeal swabs were taken prior to viral challenge (day 0) and daily for seven days. qRT-PCR was performed on each nasopharyngeal swab as described above, and non-quantitative virus detection was also done on each swab by injection into embryonated eggs as described before (Chambers et al., 2001). Infectious virus content of the nasopharyngeal swab samples from day 2 and day 3 post-challenge was determined by EID_50_ titration.

## 3 ELISA

For the evaluation of the virus-specific antibodies levels present in the sera of vaccinated mice, ELISAs were performed as previously described (Nogales et al., 2016a, Nogales et al., 2017, Nogales et al., 2016b, Rodriguez et al., 2017a, Rodriguez et al., 2017c). Briefly, 96-well plates were coated with cell lysates from mock- or EIV-infected MDCK cells and incubated overnight (O/N) at 4°C. Animal sera were assayed as two-fold dilutions (starting dilution of 1:100) and titers determined as described previously.

### 3.1 HAI assay

To evaluate the presence of EIV neutralizing antibodies, mouse sera were treated with receptor-destroying enzyme (RDE; Denka Seiken) for 16 h at 37°C and heat inactivated for 30 min at 56°C. The sera were then serially 2-fold diluted (starting dilution of 1:50) in 96-well V-bottom plates and mixed 1:1 with 4 hemagglutinating units (HAU) of A/equine/Ohio/1/2003 H3N8 during 30 min at RT. The HAI titers were determined by adding 0.5% turkey red blood cells to the virus-antibody mixtures for 30 min on ice (Nogales et al., 2016b). The geometric mean titers and SDs from individual mice (N = 6) were calculated from the last well where hemagglutination was inhibited. HAI for equine sera was performed in essentially the same manner except that equine sera were pre-treated with trypsin-periodate as described (Chambers and Reedy, 2014) to remove non-specific inhibitors of hemagglutination, and chicken red blood cells were used.

## 4 Results

### 4.1 Generation and characterization of A/equine/Ohio/1/2003 H3N8 (EIV) LAIV

**Fig. 1:**
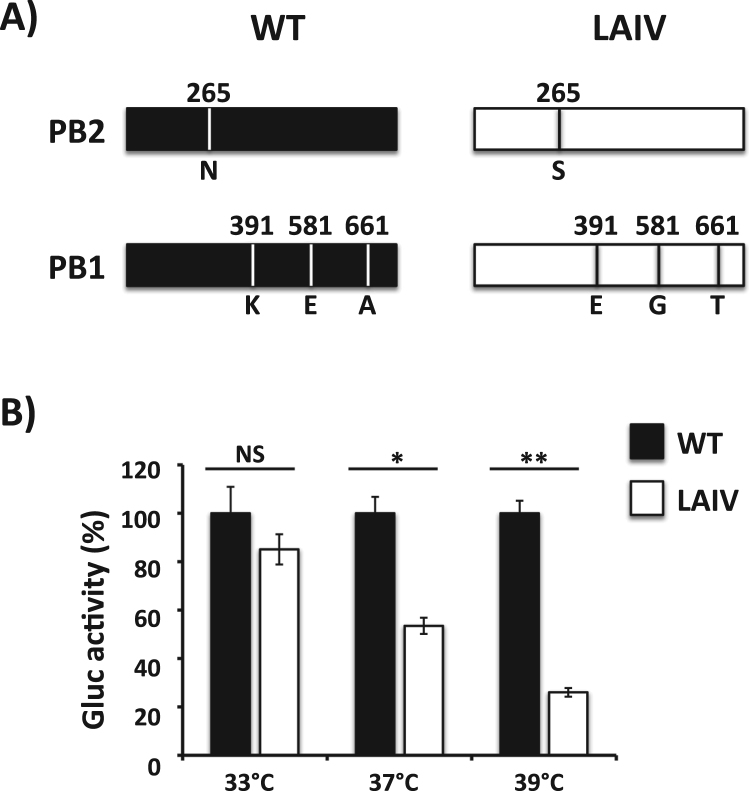
**Effect of temperature on the polymerase activity of A/equine/Ohio/1/2003 H3N8 (EIV) live-attenuated influenza vaccine (LAIV). A) Schematic representation of segments 1 (PB2) and 2 (PB1) of WT (black) and LAIV (white) EIV (A/Equine/Ohio/1/2003)**: Amino acid substitutions in the polymerase PB2 (N265S) and PB1 (K391E, E581G, and A661T) subunits of A/equine/Ohio/1/2003 H3N8 are indicated. **B) Minigenome activity**: E. Derm cells (12-well plate format, 5 × 10^5^ cells/well, triplicates) were transiently co-transfected with 0.25 μg of ambisense pDZ expression plasmids encoding the minimal requirements for viral genome replication and gene transcription (PB2, PB1, PA and NP), together with 0.5 μg of a vRNA-like expression plasmid encoding Gaussia luciferase (Gluc), and 0.1 μg of a pCAGGS Cypridinia luciferase (Cluc) plasmid to normalize transfection efficiencies. Six hours after transfection, cells were placed at 33°C, 37°C or 39°C, and 48 h post-transfection, viral replication and transcription were evaluated by luminescence (Gluc). Gluc activity was normalized to that of Cluc. Data represent the means ± SDs of the results determined for triplicate assays. Normalized reporter expression is relative to minigenome activity in the absence of the pDZ NP plasmid. Data are represented as relative activity considering WT EIV polymerase activity at each temperature as 100%. *, P < 0.005; **, P < 0.001; NS not statistical using the Student T test.

The commercially available EIV LAIV (Flu Avert I.N.) is made of an EIV strain that circulated over 25 years ago (A/equine/Kentucky/1/1991 H3N8) and has never been updated (Youngner et al., 2001). In order to generate an updated EIV LAIV, we introduced four of the five mutations responsible for the ts, ca and att phenotypes of the human A/Ann Arbor/6/60 H2N2 LAIV (FluMist) (Cox et al., 1988, Snyder et al., 1988) into the PB2 (N265S) and PB1 (K391E, E581G, A661T) genes of A/equine/Ohio/1/2003 H3N8 (EIV) (Fig. 1**A**), a clade 1 Florida sublineage strain recommended by the OIE to be included in the EIV vaccine (Paillot et al., 2016). The A/equine/Ohio/1/2003 H3N8 NP viral segment already contains a G in position 43. We then performed a minigenome replication assay in E. Derm cells at different temperatures (33°C, 37°C or 39°C) to analyze if the mutations introduced into the PB2 and PB1 genes of A/equine/Ohio/1/2003 H3N8 conferred a ts phenotype to the viral polymerase complex. At 33°C, both A/equine/Ohio/1/2003 H3N8 WT and LAIV polymerases induced similar levels of Gluc expression (Fig. 1**B**). However, Gluc expression was significantly reduced at 37°C and even more at 39°C (Fig. 1**B**).

**Fig. 2:**
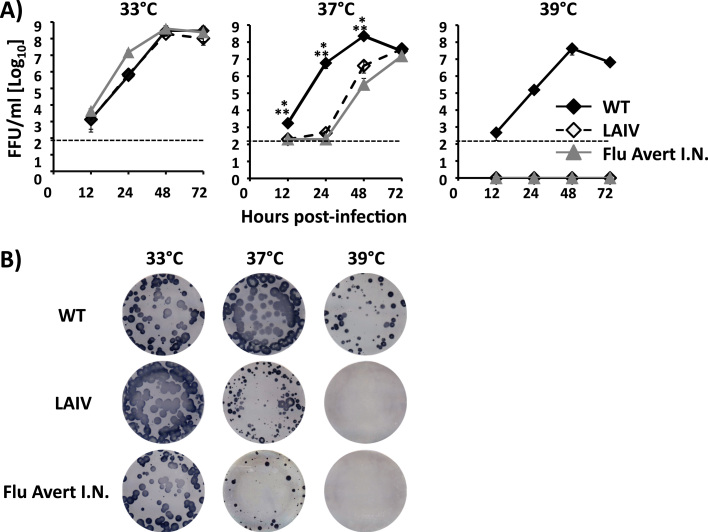
***In vitro*****characterization of EIV LAIV. A) Multicycle growth kinetics:** MDCK cells (12-well plate format, 5 × 10^5^ cells/well, triplicates) were infected (MOI, 0.001) with A/equine/Ohio/1/2003 H3N8 WT (black diamonds) and LAIV (white diamonds) and incubated at 33°C, 37°C and 39°C. As internal control, MDCK cells were also infected with Flu Avert I.N. (grey triangles). Viral titers in TCS at the indicated times post-infection were determined by immunofocus assay (FFU/ml) using an anti-NP mAb (HB-65). Data represent the means +/- SDs of the results determined in triplicate wells. Dotted black lines indicate the limit of detection (200 FFU/ml). P < 0.05: * WT vs. LAIV, ** WT vs. Flu Avert I.N. using the Student T test. **B) Plaque phenotype:** MDCK cells (6-well plate format, 1 × 10^6^ cells/well) were infected with A/equine/Ohio/1/2003 H3N8 WT and LAIV and overlaid with media containing agar. MDCK cells infected with Flu Avert I.N. were included as internal control. Plates were incubated at 33°C, 37°C and 39°C and three days p.i., monolayers were immunostained with an anti-NP mAb (HB-65).

Based on the ts phenotype observed in our minigenome assay (Fig. 1), we next assessed if the introduced mutations in the viral PB2 and PB1 polymerase subunit of A/equine/Ohio/1/2003 H3N8 would result in a virus with impaired growth kinetics at restrictive (37–39°C) but not at permissive (33°C) temperatures. Thus, we rescued WT and LAIV A/equine/Ohio/1/2003 H3N8 (referred to henceforth as EIV WT and EIV LAIV, respectively) using previously described reverse-genetic techniques (Martinez-Sobrido and Garcia-Sastre, 2010, Nogales et al., 2014b). The viral replication kinetics of both viruses were determined by evaluating viral titers in MDCK cells infected at low (0.001) multiplicity of infection (MOI) at different temperatures (33°C, 37°C or 39°C) (Fig. 2**A**). Flu Avert I.N. was also included as a control. At 33°C, both EIV WT and LAIV, and Flu Avert I.N., grew with similar kinetics and reached peak titers at 48 h p.i. At 37°C and 39°C, EIV WT replication was similar to that observed at 33°C. The titers of EIV LAIV and Flu Avert I.N. were significantly reduced or not detected at 37°C and 39°C, respectively, as compared to EIV WT (Fig. 2**A**). We also analyzed the plaque phenotype of EIV WT and LAIV, and Flu Avert I.N. at the same temperatures (33°C, 37°C or 39°C) (Fig. 2**B**). EIV WT plaque size was similar at 33°C and 37°C, and slightly reduced at 39°C in accordance with the minimal reduction in viral titers observed in the kinetics at that temperature (Fig. 2**A**). In the case of EIV LAIV and Flu Avert I.N., the size of the plaques at 33°C was similar to that of EIV WT, but at high temperatures, the plaque size was strongly reduced (37°C) or plaques were not detected (39°C), corroborating the growth kinetic results (Fig. 2**A**). Altogether, these results demonstrate that amino acid substitutions in the PB2 and PB1 polymerase subunits of A/equine/Ohio/1/2003 H3N8 confer a ts phenotype similar to that observed in the human A/Ann Arbor/6/60 H2N2 LAIV (Chan et al., 2008), These characteristics have also been previously described for other influenza A viruses (Cox et al., 2015, Jin et al., 2004, Nogales et al., 2016b, Rodriguez et al., 2017c, Zhou et al., 2012).

### 4.2 Attenuation of EIV LAIV in mice

**Fig. 3:**
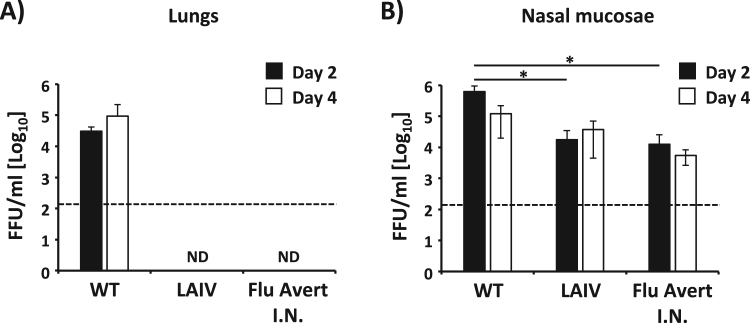
**Attenuation of EIV LAIV in mice:** Female 6-to-8-week-old C57BL/6 mice (N = 6) were infected intranasally (i.n.) with 1 × 10^5^ FFU of A/equine/Ohio/1/2003 H3N8 WT or LAIV. Mice were also infected with 1 × 10^5^ FFU with Flu Avert I.N. as internal control. Presence of viruses in lungs (**A**) and nasal mucosa (**B**) of infected mice were evaluated at days 2 (N = 3) and 4 (N = 3) p.i. by immunofocus assay (FFU/ml) using an anti-NP mAb (HB-65). Data represent the means ± SDs. Dotted black lines indicate the limit of detection (200 FFU/ml). ND, not detected. *, P < 0.05 using the Student *T* test.

After elucidating that the growth kinetics (Fig. 2**A**) and the plaque size (Fig. 2**B**) of EIV LAIV were affected at high temperatures (37°C and 39°C) but not at low temperatures (33°C), we next analyzed its ability to replicate *in vivo* in a mouse model of influenza infection (Fig. 3). To that end, mice (N = 3/time point) were infected i.n. with 10^5^ FFU of EIV WT or LAIV. Mice were also infected with 10^5^ FFU of Flu Avert I.N. as an internal control. Since no signs of infection were detected in mice after infection with EIV WT, replication of EIV WT and LAIVs were determined by evaluating viral titers from the lungs (Fig. 3**A**) and nasal mucosa (Fig. 3**B**) at days 2 and 4 p.i. We decided to use this high dose (10^5^ FFU) to better evaluate the safety profile of the new EIV LAIV in comparison with its WT counterpart. Notably, viral titers were only detected in the lungs of mice infected with EIV WT at both times p.i. (Fig. 3**A**), but no virus was detected in the lungs of mice infected with EIV LAIV or Flu Avert I.N. (Fig. 3**A**). On the other hand, viral replication was detected in the nasal mucosa of mice infected with the three viruses (Fig. 3**B**), although the viral titers obtained in mice infected with EIV LAIV and Flu Avert I.N. were significantly lower at both times p.i. as compared to EIV WT. These results indicate that our EIV LAIV was also attenuated *in vivo* at high temperatures (lungs) but able to replicate in the nasal mucosa where the temperature is lower. Importantly, the same *in vivo* ts phenotype was observed with Flu Avert I.N.

### 4.3 Mice immunized with EIV LAIV are protected against H3N8 EIV WT challenge

**Fig. 4:**
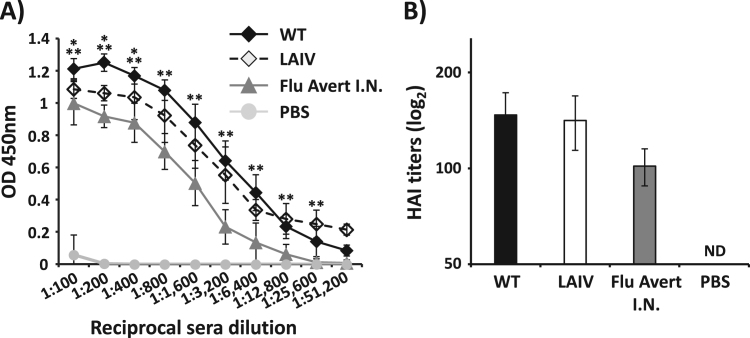
**Induction of humoral responses by EIV LAIV in mice:** Female 6-to-8-week-old C57BL/6 mice (N = 6) were vaccinated (i.n.) with 1 × 10^3^ FFU of A/equine/Ohio/1/2003 H3N8 WT or LAIV. Mice were also mock (PBS) vaccinated or vaccinated (i.n.) with 1 × 10^3^ FFU of Flu Avert I.N. as negative and positive controls, respectively. At 14 days post-vaccination, mice were bled and sera were collected and evaluated individually for the presence of total antibodies by ELISA (**A**) and neutralizing antibodies by HAI (**B**) against A/equine/Ohio/1/2003 H3N8. OD, optical density. Data represent the means +/- SDs of the results for 6 individual mice. ND, not detected. *, P < 0.05 wt vs. LAIV; **, P < 0.005 wt vs. Flu Avert I.N. using the Student *T* test.

To evaluate the immunity induced by EIV LAIV, groups of mice (N = 6) were vaccinated i.n. with 10^3^ FFU of WT and LAIV EIVs, mock vaccinated with PBS or vaccinated i.n. with 10^3^ FFU of Flu Avert I.N. as negative and positive controls, respectively. We choose the 10^3^ FFU/mouse dose because base on the safety results (Fig. 3) is a secure dose and, because in previous manuscripts in which we described the development of LAIVs against H3N8 (Nogales et al., 2016b) and H3N2 (Rodriguez et al., 2017c) CIVs, this dose induced strong humoral and cellular responses, as well as complete protection against challenge with WT CIVs. Humoral immune responses were evaluated in mouse sera collected 14 days post-vaccination. Antibody responses against total EIV proteins were evaluated by ELISA using cell extracts from virus-infected MDCK cells (Fig. 4**A**) (Nogales et al., 2016b, Rodriguez et al., 2017c). Sera from mice vaccinated with EIV LAIV elicited high serum IgG titers against EIV proteins, close to those obtained in the sera from mice infected with EIV WT, while a significant lower titer of antibodies was observed in the sera from mice immunized with Flu Avert I.N. (Fig. 4**A**). Additionally, we performed HAI assays to evaluate the presence of neutralizing antibodies in sera from vaccinated mice (Fig. 4**B**). HAI titers against EIV were higher in the sera from mice vaccinated with EIV LAIV than those observed in mice vaccinated with Flu Avert I.N and were similar to those obtained in EIV WT infected mice (Fig. 4**B**).

**Fig. 5:**
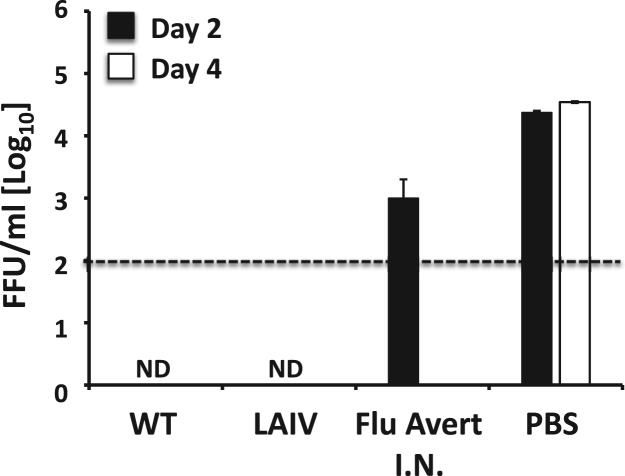
**Protection efficacy of EIV LAIV against EIV challenge in mice:** Female 6- to-8-week-old C57BL/6 mice (N = 6) were vaccinated with 1 × 10^3^ FFU of A/equine/Ohio/1/2003 H3N8 WT or LAIV. Mice were also mock (PBS) vaccinated or vaccinated (i.n.) with 1 × 10^3^ FFU of Flu Avert I.N. as negative and positive controls, respectively. At 15 days post-vaccination, mice were challenged with 1 × 10^5^ FFU of A/equine/Ohio/1/2003 H3N8 WT and viral titers at days 2 (N = 3) and 4 (N = 4) post-challenge were evaluated from lung homogenates by immunofocus assay (FFU/ml) using an anti-NP mAb (HB-65). Dotted black line indicates the limit of detection (200 FFU/ml). Data represent the means ± SDs. ND, not detected.

Next, we evaluated the protection efficacy induced by our EIV LAIV against homologous A/equine/Ohio/1/2003 H3N8 WT challenge (Fig. 5). Mice (N = 6) were vaccinated i.n. with 10^3^ FFU of WT and LAIV EIVs, Flu Avert I.N., or mock vaccinated with PBS. Fifteen days after vaccination, mice were challenged with 10^5^ FFU of A/equine/Ohio/1/2003 H3N8 WT and viral titers in the lungs of infected mice (N = 3 / group) were determined 2 and 4 days after challenge (Fig. 5). Mock-vaccinated (PBS) mice exhibited lung viral titers of ~ 3 × 10^4^ FFU/ml at days 2 and 4 post-challenge, whereas mice vaccinated with WT or LAIV EIVs showed no detectable virus in the lungs at those times (Fig. 5). Contrarily, A/equine/Ohio/1/2003 H3N8 WT was detected in the lungs of mice vaccinated with Flu Avert I.N. at day 2 post-challenge (~ 1 × 10^3^ FFU/ml), but not at day 4 post-challenge (Fig. 5). These results indicate that our EIV LAIV induced better protection than Flu Avert I.N. against a challenge with A/equine/Ohio/1/2003 H3N8 WT in mice, probably because of the antigenic match.

### 4.4 Attenuation of EIV LAIV in horses

**Fig. 6:**
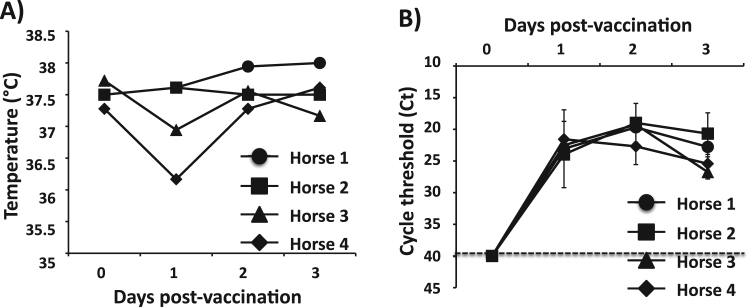
**Attenuation of EIV LAIV in horses:** One-to-two years-old horses of both sexes (N = 4) were inoculated i.n. with 4 × 10^8^ FFU of A/equine/Ohio/1/2003 H3N8 LAIV. **A**) Graphic representation of the individual rectal temperatures measured in each horse before (day 0) and during 3 days after vaccination. **B**) The virus content in nasopharyngeal swabs were determined by quantitative (q)RT-PCR and represented as quantification cycle threshold (Ct). The swabs were taken before (day 0) and during 3 days post-vaccination for each horse nostril. Data represent the means from each horse in each time post-vaccination ± SDs. Dotted black line indicates the limit of detection (Ct = 40).

**Fig. 7:**
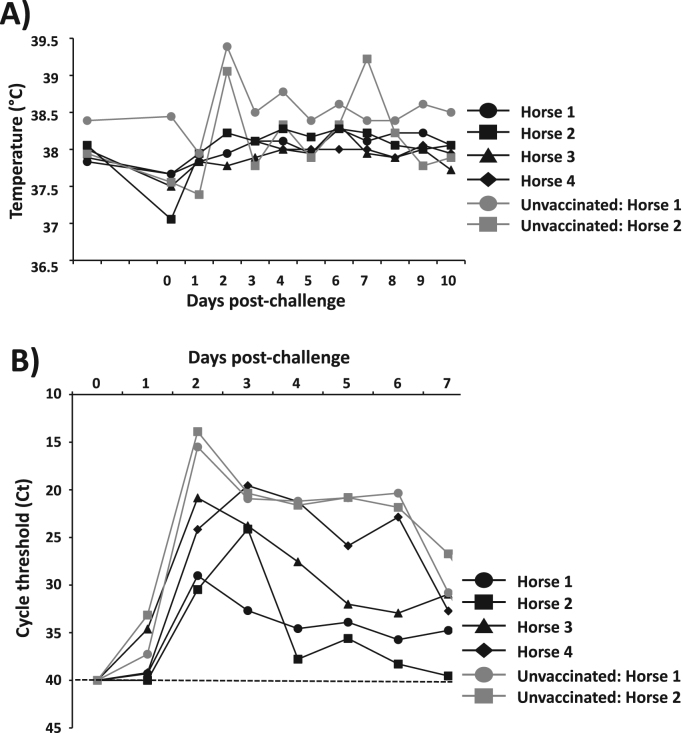
**Protection efficacy of EIV LAIV against EIV challenge in horses:** One-to-two years-old horses of both sexes (N = 4) were vaccinated by i.n. intubation with 4 × 10^8^ FFU of A/equine/Ohio/1/2003 H3N8 LAIV. Another group of horses (N = 2) were used as a control (unvaccinated). At 27 days post-vaccination, horses were challenged by aerosolized with 1 × 10^7^ EID50 units per m^3^ into a tented stall (37.5 m^3^) for 45 min. **A**) Rectal temperatures were measured daily by 10 days after challenge. **B**) Virus content in nasopharyngeal swabs taken during 7 days post-challenge was analyzed by (q)RT-PCR and represented as cycle threshold (Ct). Dotted black line indicates the limit of detection (Ct = 40).

We next evaluated the safety as well as the protection efficacy induced by our EIV LAIV in horses, its natural host. To this end, four horses were infected i.n. with 4 × 10^8^ FFU of EIV LAIV and monitored for clinical signs such as cough, nasal discharge, respiration and depression, rectal temperature as well as viral shedding during the first 3 days after infection (Fig. 6). None of the horses showed significant adverse effects. Three of the four horses showed a slight, bilateral serous nasal discharge at days 2 and 3 p.i. and a single incidence of coughing was observed (data not shown), however rectal temperatures remained normal (37.5°C ± 0.2 on day of vaccination, 37.6°C ± 0.4 on Day + 3) (Fig. 6**A)**. To measure the presence of EIV LAIV in nasopharyngeal swabs collected at days 0–3 p.i., a qRT-PCR was performed on each swab (one swab for each nostril of each horse per day). Virus shedding was detected in all nasopharyngeal swabs collected on days 1–3 p.i. showing a peak at day 2 p.i. (Fig. 6**B**), indicative of viral replication. The horses were observed daily for an additional 25 days although further swabbing past day 3 p.i. to ascertain the duration of shedding was not done. During that period, one horse was euthanized for an unrelated problem (equine protozoal myelitis). Similar safety observations, including slight serous nasal discharge in 2/4 horses, were obtained from the yearling horses that were subsequently challenged (Fig. 7). Following vaccination, all horses showed seroconversion as their HAI antibody titers increased from undetected (< 10) to 20 (in three horses of both the safety and challenge trials) or 10 (in the 4th horse of both trials) and, as expected, no HAI antibodies were detected in the sera from the unvaccinated control group. These results demonstrate the safety profile of our EIV LAIV in horses and their ability to replicate in the upper respiratory track, necessary for the induction of immunity, including HA-specific antibody responses.

### 4.5 Horses immunized with EIV LAIV are protected against challenge with heterologous EIV H3N8 WT

In order to evaluate the protection efficacy induced by our EIV LAIV in its natural host, a group of horses (N = 4) was vaccinated as previously indicated with 4 × 10^8^ FFU of EIV LAIV, or mock vaccinated (N = 2), as negative control (Fig. 7). Twenty-seven days after vaccination, horses were challenged by aerosolized with 1 × 10^7^ EID_50_ units per m^3^ of A/equine/Kentucky/2014 H3N8 WT into a tented stall (37.5 m^3^) for 45 min. A/equine/Kentucky/14 (H3N8) virus, a Florida clade 1 strain is heterologous yet antigenically similar to our EIV LAIV. During the first 10 days after challenge, horses were monitored for rectal temperatures (Fig. 7**A**), presence of clinical symptoms of infection (cough, nasal discharge, respiration, depression and swelling of lymph nodes) and virus shedding (Fig. 7**B**). Both unvaccinated controls, but none of the four horses vaccinated with EIV LAIV exhibited a characteristic spike of pyrexia on day two post-challenge (Fig. 7**A**), and also one of the unvaccinated horses (number 2) was noted as lethargic on day two post-challenge. Body temperatures of the two control horses returned to normal or near-normal range on days three to six post-challenge, but the unvaccinated horse number 2 had a second fever spike on day seven post-challenge (Fig. 7**A**). Both unvaccinated horses had cough on days three (horse number 2) and seven (horse number 1) different days post-challenge, while coughing was not observed in any of the vaccinates. Nasal discharge was observed in both control animals on day eight (unvaccinated horse 1) or day two (unvaccinated horse 2) post-challenge. Notably, none of the vaccinated horses had cough or nasal discharge. Another clinical symptom observed in the unvaccinated horses was inspiratory wheeze on day six (unvaccinated horse 1) and day four (unvaccinated horse 2) post-challenge, but not in the vaccinated horses. Swelling of submandibular or parotid lymph nodes was observed in three out of four vaccinates as well as both controls; however, the severity and duration were greater in the controls. Late in the study (at day 11 post-challenge) an independent veterinary assessment led to both control horses, but none of the vaccines, being treated with antibiotics to promote full recovery. From a clinical standpoint, therefore, vaccinated horses appeared to be protected from challenge with wild-type EIV.

A/equine/Kentucky/2014 H3N8 WT virus shedding in nasopharyngeal swabs was evaluated by inoculation of embryonated chicken eggs and also by direct qRT-PCR (Fig. 7**B**). When the nasopharyngeal swabs from vaccinated horses were inoculated in eggs, live virus was detectable at least one time post-challenge, with an average of 2.25 days up to maximum of five days post-challenge. EID_50_ titrations of infectious virus content in the swab material collected at day two or three post-challenge showed titers between 1.7 × 10^2^ and 3.16 × 10^3^ EID_50_ units/ml. On the other side, both unvaccinated horses shed detectable live virus for five and six days post-challenge, and viral titers in the allantoic fluid at two days post-inoculation were 1.7 × 10^5^ (number 2) and 4.6 × 10^7^ (number 1) EID_50_ units/ml. Thus, our EIV LAIV did not achieve sterilizing immunity against an heterologous challenge after a single dose, but live virus shedding appeared to be reduced by at least three orders of magnitude comparing with the unvaccinated horses. These results were confirmed when we evaluated the presence of virus by qRT-PCR in the daily nasopharyngeal swabs (Fig. 7**B**). In both horses’ groups (vaccinated or unvaccinated) there was detectable virus amplification continuously from day one post-challenge (or day two for the vaccinated horse 2) through day seven when swabbing was discontinued. The peaks shedding in unvaccinated horses were greater than the values obtained in vaccinated horses with a difference between 5 and 15 cycles suggesting about 500 to 1500 times greater target concentration than in vaccinated horses. By 14 days following viral challenge, all horses exhibited 16–32-fold increases in serum HAI antibody titers. Altogether, the results show that our EIV LAIV induced protection against a heterologous challenge whit A/equine/Kentucky/2014 H3N8 WT.

## 5 Discussion

Equine influenza, currently caused by H3N8 EIV, is the most common and important respiratory infectious disease of horses (Daly et al., 2011, Timoney, 2000). H3N8 EIV is highly contagious and has the potential to spread rapidly through groups of naive horses in aerosolized droplets that are dispersed by coughing (Daly et al., 2011, Timoney, 2000). H3N8 EIV infections of horses have been responsible for disrupting major equestrian events and causing significant economic losses (Daly et al., 2011, Timoney, 2000). The equine population is highly mobile, and horses travel long distances by road and/or air for competitions and breeding purposes. When an infected horse is introduced into a susceptible population, the spread of H3N8 EIV can be explosive. Large outbreaks of H3N8 EIV are often associated with the congregation of horses at equestrian events. Their dispersal after these events can lead to further widespread dissemination of the virus. It is currently estimated that H3N8 EIV outbreaks result in economic losses of hundreds of millions of dollars. In endemic countries, the significant economic losses caused by H3N8 EIV infections can be minimized by vaccination of highly mobile horses. Indeed, many racing and equestrian authorities have mandatory vaccination policies that serve as insurance for business. On the other hand, non-endemic countries rely on vaccination of imported horses and quarantine to prevent an incursion of H3N8 EIV. The majority of these non-endemic countries also require vaccination of their indigenous horse population to reduce the potential impact of an H3N8 EIV incursion.

Traditional vaccination strategies support that vaccine strains must represent viruses in circulation, and it is only through surveillance that vaccine companies decide on which antigens should be used. Thus, EIV surveillance and strain characterization are fundamental for H3N8 EIV control programs based on vaccination. Importantly, vaccine manufacturers need to have a dynamic vaccination approach that allows the rapid generation of novel vaccines to benefit the equine population (Cullinane et al., 2010, Paillot, 2014, Paillot et al., 2016). Results from cross-protection studies indicate that the majority of the inactivated vaccines or the current commercially available LAIV Flu Avert I.N. would provide poor levels of protection if used in the face of an imminent outbreak because of the antigenic differences between the virus in the vaccine and currently circulating H3N8 EIV strains (Paillot et al., 2016). Notably, some recent H3N8 EIV outbreaks occurred in previously vaccinated animals, where the vaccine strain did not match the circulating virus (Daly et al., 2003, Garner et al., 2011, Timoney, 2000). The frequency of H3N8 EIV outbreaks, the continuous antigenic variation (antigenic drift) of H3N8 EIV and examples of vaccine breakdown due to poorly antigenic match demonstrate the periodic need to update EIV vaccines to prevent equine influenza in the equine population. Moreover, EIV vaccines should include both clade 1 and clade 2 representative strains of the Florida sublineage, as recommended by the OIE (Paillot et al., 2016).

Here, we report the development of a novel and more effective LAIV for the prevention and control of equine influenza using reverse genetics. This is the first time than an i.n. competitive ts LAIV based on reverse genetic techniques has been developed for the prevention and control of H3N8 EIV in horses. To generate our H3N8 EIV LAIV, we introduced in the PB2 and PB1 viral genes from A/equine/Ohio/1/2003 H3N8, a strain recommended by the OIE to be part of EIV vaccines (clade 1 of Florida sublineage) (OIE, 2017), the mutations responsible for the ca, ts and att phenotypes of the human MDV A/Ann Arbor/6/60 H2N2 LAIV (Cox et al., 1988, Snyder et al., 1988) (Fig. 1). *In vitro*, the recombinant A/equine/Ohio/1/2003 H3N8 LAIV (EIV LAIV) replicated efficiently at low temperature (33°C), which is important for vaccine production, but was restricted in replication at higher (37°C and 39°C) temperatures, imperative for its safe implementation as LAIV (Fig. 2). In a mouse model of influenza infection, our EIV LAIV was attenuated in the lower respiratory tract (lungs) but not in the upper respiratory tract (nasal mucosa) when compared to its WT counterpart (Fig. 3). Importantly, the phenotype observed with our EIV LAIV *in vivo* and *in vitro* was the same as that observed with the currently available H3N8 EIV LAIV, Flu Avert I.N. Notably, our EIV LAIV was able to induce, upon a single i.n. immunization dose, complete protection against challenge with A/equine/Ohio/1/2003 H3N8 WT, contrary to Flu Avert I.N. that only showed partial protection (Fig. 5)**.** This partial protection observed with Flu Avert I.N. is probably due to the fact that Flu Avert I.N. is based on a virus that is antigenically distant from current EIV circulating strains, including that used in our study (A/equine/Ohio/1/2003). The analysis of humoral responses showed that the titer of total (Fig. 4**A**), as well as neutralizing (Fig. 4**B**), antibodies against A/equine/Ohio/1/2003 H3N8 WT was higher in sera from mice immunized with our EIV LAIV than in sera from mice vaccinated with Flu Avert I.N. In horses, its natural host, our EIV LAIV was safe since horses did not develop any symptoms of infection including fever (Fig. 6**A**), and was able to replicate in the upper respiratory track since the virus was detected in nasal swabs (Fig. 6**B**), where the temperatures is low, which is essential to induce mucosal immunity. Serum antibody titers in horses following vaccination were low, which was also reported with the Flu Avert I.N. LAIV in horses following a single dose (Lunn et al., 2001, Townsend et al., 2001). Those authors argued that other indices of immunological response, such as local mucosal immunity, appear to be more relevant than serum antibody levels (Lunn et al., 2001, Townsend et al., 2001). Importantly, in the horse vaccination and challenge experiment with the heterologous A/equine/Kentucky/2014 H3N8 WT virus (Florida clade 1 strain), none of the horses vaccinated with our EIV LAIV showed clinical symptoms of infection after challenge, with the exception of swelling of submandibular or parotid lymph nodes but with a lower severity and duration than the observed in unvaccinated horses. It is true than in all horses (vaccinated or unvaccinated) the challenged A/equine/Kentucky/2014 H3N8 WT virus was detected in nasopharyngeal swabs by qRT-PCR (Fig. 7**B**) and by growth in embryonated chicken eggs, but in both systems the virus detected was three orders of magnitude lower in vaccinated horses. All these results indicate that our EIV LAIV induces protection against a A/equine/Kentucky/2014 H3N8 WT heterologous challenge, even though it did not induce sterilizing immunity. One possibility of this not complete protection could be the need for a two-dose regime vaccination in naïve horses. Another possibility could be a mismatch between both EIV H3N8 viruses.

Compared to current H3N8 EIV IIVs, our H3N8 EIV LAIV approach presents several advantages. First, our H3N8 EIV LAIV is administered intranasally and mimics the natural route of viral infection, therefore inducing mucosal immune responses at the site of infection (Kohlmeier and Woodland, 2009, Murphy and Coelingh, 2002). Second, a significant lower amount of virus in our H3N8 EIV LAIV is required to induce superior protection than that required with H3N8 EIV IIVs (Nogales et al., 2016b, Rodriguez et al., 2017c). Third, LAIVs have been shown to stimulate more robust systemic humoral response (Cheng et al., 2013, De Villiers et al., 2009, Katsura et al., 2012, Nogales et al., 2016b, Rodriguez et al., 2017c, Victor et al., 2012) and elicit cellular immunity (Cheng et al., 2013, Katsura et al., 2012), leading to recruitment of influenza-specific CD8 T cells in the target tissues of the respiratory tract (Baker et al., 2013, Guo et al., 2014, Katsura et al., 2012, Nogales et al., 2016b, Powell et al., 2012, Rodriguez et al., 2017c, Uraki et al., 2013). Fourth, a single immunization with our H3N8 EIV LAIV would be sufficient to confer at least partial protection against H3N8 EIV WT in a shorter period of time, compared with the multiple doses required with the current inactivated vaccines. Finally, our H3N8 EIV LAIV would provide better cross protection against antigenically distinct H3N8 EIV strains than that provided by EIV IIVs, diminishing the chance of EIV outbreaks. Some of the above advantages are shared by the only commercially available H3N8 EIV LAIV, Flu Avert I.N. (Chambers et al., 2001). However, our technology also offers a number of additional advantages. First, the mutations introduced in the PB2 and PB1 polymerase subunits of A/equine/Ohio/1/2003 H3N8 have been previously described to be responsible for the ts, ca and att phenotype in the MDV of the human A/Ann Arbor/6/60 H2N2 LAIV (FluMist) (Cox et al., 1988, Snyder et al., 1988) which have a proven history of safety, immunogenicity and protection efficacy not only against human viruses, but also against avian and equine influenza viruses (Baz et al., 2015, Suguitan et al., 2006). Second, same ts and ca mutations were also introduced in other influenza A viruses inducing the same attenuated phenotype (Cox et al., 2015, Jin et al., 2004, Nogales et al., 2016b, Rodriguez et al., 2017c, Zhou et al., 2012). Third, the use of state-of-the-art reverse genetic techniques will facilitate, similar to the case of the human LAIV, the fast and accurate development of LAIV candidates for the control of currently circulating clades 1 and 2 strains of the Florida sublineage, or newly introduced EIV strains in the case of a new outbreak in the horse population. To that end, our temperature sensitive A/equine/Ohio/1/2003 H3N8 LAIV could be used as a MDV to produce updated LAIV by the introduction of HA and NA from antigenically different circulating H3N8 EIV strains or newly introduced EIVs in the horse population, including EIVs with panzootic potential. Finally, our approach could be updated to develop a bivalent H3N8 EIV LAIVs that follow the current OIE recommendations to include representative strains of the clades 1 and 2 of Florida sublineages of H3N8 EIVs. We have recently used a similar strategy to develop a bivalent LAIV for the control of H3N8 and H3N2 canine influenza virus infections (Rodriguez et al., 2017b). Based on the multiple advantages over H3N8 EIV IIVs or the current LAIV, our novel platform represents an easier and faster approach for the feasibility of implementing a safe and more effective LAIV for the prevention and control of H3N8 EIVs in the equine population, reducing the burden of current and future influenza disease in horses.
